# Reviewed article: Rosa RS, Menezes JS, Martinez EZ, Boery RNSO, Ribeiro IJS, Contreras JCZ, Junior JB, Mussi RFF, Moreira SLF, Lemos GS, Santos ISC. Factors associated with inadequate blood pressure control among hypertensive individuals living in a Quilombola Community: a cross-setional study, Brazil, 2017-2018. Epidemiol Serv Saude. 2025:34;e20240556

**DOI:** 10.1590/S2237-96222025v34e20240556.a

**Published:** 2025-10-03

**Authors:** Kaciane Corrêa Mochizuke

**Affiliations:** 1 Prefeitura Municipal de Porto Murtinho, Porto Murtinho, MS, Brazil Prefeitura Municipal de Porto Murtinho Porto Murtinho MS Brazil

## Peer review A

## Questionnaire

Does the manuscript contain new and significant information to justify publication? Yes

Does the Abstract (Summary) clearly and accurately describe the content of the article? No

Is the problem significant and concisely stated? No

Are the methods described comprehensively? No

Are the interpretations and conclusions justified by the results? Yes

Is adequate reference made to other work in the field? No

## Manuscript Structure

Length of article is: Adequate

Number of tables is: Too few

Number of figures is: Too few

Please state any conflict(s) of interest that you have in relation to the review of this paper. None

Interest: Good

Quality: Below Average

Originality: Good

Overall: Below Average

Recommendation: Minor Revision

## Comments

Sugiro revisão do título do manuscrito, informando ano da pesquisa, em consonância com as instruções aos autores.

O objetivo do resumo pesquisa precisa ser mais bem definido e claro: “identificar a prevalência e fatores associados ao controle pressórico em hipertensos residentes em uma comunidade quilombola” O desfecho controle pressórico não é compreensível. Sugiro observar a seguinte instrução da RESS: ”Certifique-se de que a estrutura da frase faça sentido lógico semanticamente, evitando construções inconsistentes ou paradoxais, como "presença de ausência". Seja particularmente cuidadoso com desfechos, especialmente os negativos. Isso se aplica tanto à escolha do termo para sua designação (por exemplo, "nenhum exame do pé" pode ser "negligência no exame do pé") quanto a declarações que envolvam termos negativos (por exemplo, "a presença de negligência foi maior em idosos" pode ser "a negligência foi maior em idosos"). Esforce-se para uma comunicação clara que transmita informações compreensíveis”.

Sugiro rever o delineamento do estudo, com a correção da definição do tipo do estudo e da sua população. Onde cita “estudo epidemiológico, censitário, de delineamento transversal e de base comunitária” torne-o mais objetivo e condizente com sua amostra. Termos como “transversal de base comunitária”, “censitário” indicam que foi feito um levantamento com informações de todas as pessoas de um determinado grupo, contudo sua amostra é limitada a pessoas com “cadastro na Unidade de Saúde da Família” configurando uma amostra por conveniência que não representa uma base comunitária. A partir dos resultados apresentados sugere-se que o denomine como estudo transversal.

A metodologia não descreve quando e onde a aplicação dos questionários e a avaliação da composição corporal e aferição da pressão ocorreu. Descreva os métodos com mais detalhes, incluindo o processo de coleta e análise de dados, para que outros pesquisadores possam replicar o estudo.

Descreva o instrumento de coleta de dados, incluindo informações sobre sua validação e confiabilidade. Se o instrumento não foi validado, discuta as limitações dessa falha. Há inconsistência, por exemplo no resultado da variável Diabetes tipo e tipo 2. Como isso foi medido? Pelos resultados apresentados há pessoas com os dois tipos de diabetes. Por outro lado, qual seria relevância de trabalhar os dois tipos de diabetes, uma vez que os dados não são claros e sua coleta não apresenta confiabilidade.

Remova informações irrelevantes: “a "Avaliação clínica do risco cardiovascular e fatores associados a adesão terapêutica de pessoas hipertensas em famílias quilombolas”.

Relate a proporção de perdas de participantes e/ou a taxa de resposta, discutindo o impacto potencial dessas perdas nos resultados do estudo, sugiro adição de um gráfico de fluxo para esclarecer a seleção da amostra.

Revise o uso de siglas, restringindo-as às consagradas e ao mínimo essencial para a fluidez do texto: ”e aferição da PA para a avaliação clínica”, referindo-se apenas à pressão.

Reveja os títulos das tabelas. Tendo em mente que os títulos devem ser autossuficientes para a ilustração, dispensando consultar o texto. Siglas essenciais para compreensão da ilustração devem constar preferencialmente no título, conforme exemplo: “Tabela 3. Razões de prevalências (RP) e intervalos de confiança de 95% (IC95%) brutas e ajustadas do [desfecho] pelas variáveis do estudo. Local, ano (n = xx)”. Sugiro remoção das notas de rodapé.

Busque referências bibliográficas atuais, como preconizado pela revista. Mais de 50% das suas citações possuem mais de 5 anos.

